# Neuropathic pain and use of Pain*DETECT* in patients with fibromyalgia: a cohort study

**DOI:** 10.1186/1471-2377-13-21

**Published:** 2013-02-14

**Authors:** Jarno Gauffin, Tiina Hankama, Hannu Kautiainen, Pekka Hannonen, Maija Haanpää

**Affiliations:** 1 ORTON Rehabilitation Centre, ORTON Foundation, Helsinki, Finland; 2 Department of Medicine, Central Finland Central Hospital, Jyväskylä, Finland; 3 Unit of Family Practice, Central Finland Central Hospital, Jyväskylä, Finland; 4 Unit of Primary Health Care, Kuopio University Hospital, Kuopio, Finland; 5 University of Eastern Finland, School of Medicine, Kuopio, Finland; 6 Etera Mutual Pension Insurance Company, Helsinki, Finland; 7 Department of Neurosurgery, Helsinki University Central Hospital, Helsinki, Finland

## Abstract

**Backround:**

Fibromyalgia has a plethorae of symptoms, which can be confusing and even misleading. Accurate evaluation is necessary when patients with fibromyalgia are treated. Different types of instruments are available for the clinicians to supplement evaluation. Our objective was to study the applicability of the Pain*DETECT* instrument to screen neuropathic pain in patients with fibromyalgia.

**Methods:**

158 patients with primary fibromyalgia underwent a neurological examination including bedside sensory testing. They also fulfilled four questionnaires: Pain*DETECT*, Beck depression inventory IA (BDI IA), Fibromyalgia Impact Questionnaire (FIQ) and a self-made questionnaire regarding present pain and pain relieving methods of the patients. The results of the clinical evaluation and questionnaires were then compared.

**Results:**

Clinically verified neuropathic pain was diagnosed in 53/158 [34% (95% Cl: 26 to 41)] patients. The ROC curve achieved a maximum Youden´s index at score of 17 when sensitivity was 0.79 (95% Cl: 0.66 to 0.89) and specificity 0.53 (95% Cl: 0.43 to 0.63). The Pain*DETECT* total score (OR: 1.14 95% Cl: 1.06 to 1.22), FM as the worst current pain (OR: 0.31; 95% 0.16 to 0.62), body mass index (BMI) (OR: 1.05; 95% Cl: 1.00 to 1.11) and the intensity of current pain (OR: 1.20; 95% Cl: 1.01 to 1.41) were significantly associated with the presence of neuropathic pain in univariate analyses.

**Conclusion:**

This study highlights the importance of thorough clinical examination. The Neuropathic pain screening tool Pain*DETECT* is not as useful in patients with fibromyalgia as in patients with uncompromised central pain control.

## Background

Fibromyalgia (FM) is a chronic pain syndrome, which affects up to 5% of the general population [1]. Characteristic features of FM are widespread musculoskeletal pain and tenderness as well as fatigue in the absence of any explanatory organic disease [2]. Other usual symptoms are disturbed sleep, cognitive problems and a variety of psychosomatic symptoms originating from various organs [3]. Patients with FM often complain also about tingling, numbness, burning pain, cutaneous hyperalgesia, and pain attacks [4], which are typical symptoms of neuropathic pain. The IASP (International Association for Study of Pain) defined neuropathic pain recently as “pain caused by a lesion or disease of the somatosensory system” [5]. The prevalence of neuropathic pain in the general population is poorly known. Two population-based studies from Europe reported the prevalence of pain predominantly of neuropathic origin [6] or pain with neuropathic characteristics [7] to be 8% and 7%, respectively when assessed with a screening questionnaire without clinical confirmation of the diagnosis. According to a population-based study, the prevalence of neuropathic pain is around 10% in citizens aged 30 years or older [8].

Neuropathic pain screening tools such as Pain*DETECT* are recommended for identifying patients with suspected neuropathic pain, particularly when used by non-specialists [9,10]. Baron et al. [11] also showed that Pain*DETECT* is useful for identifying different sensory profiles of neuropathic pain when a neuropathic pain condition (e.g. diabetic neuropathy or postherpetic neuralgia) has already been diagnosed. Pain*DETECT,* which was developed and validated in Germany, incorporates a self-report questionnaire with 9 items [12]. There are 7 weighted sensory descriptor items and 2 items relating to the spatial (radiating) and temporal characteristics of the individual pain pattern. Its sensitivity and specificity compared to clinical diagnosis is 85% and 80%, respectively. PainDETECT was initially developed and validated in patients with back pain but has shown applicability also to patients with other types of neuropatic pain. When using Pain*DETECT* for screening purposes Freynhagen et al. [12] found cut-off scores ≤ 12 (a neuropathic component is unlikely) and ≥ 19 (a neuropathic component is likely) to be most appropriate. Pain*DETECT* has been translated into several languages, including Finnish.

In this study we report the applicability of the Pain*DETECT* tool to screen neuropathic pain in patients with fibromyalgia (FM).

## Methods

### Patients

Participants for the study were recruited from the patients with FM who had been diagnosed and treated in outpatient departments of Rheumatology or Physical medicine and rehabilitation of Jyväskylä Central Hospital between 2006 and 2008. Patients were identified using the ICD-10 code M79.0 according to the 2006 version. Based on medical records, patients with previously diagnosed neuropathic pain or neuropathy, active inflammatory arthritis, systemic connective tissue disease, cognitive impairment, severe psychiatric disorders (e.g., psychotic disorder, major depression, or severe anxiety disorder diagnosed by a psychiatrist) or any other unstable disease (e.g., cancer) were excluded. Only patients aged 18–65 years were included.

### Data collection

The questionnaires and consent form were sent to all traceable patients. The patients were asked to fill in four questionnaires: (1) Pain*DETECT*[12], (2) Beck depression inventory IA (BDI IA) [13], (3) Fibromyalgia Impact Questionnaire (FIQ) [14], and (4) a one-page self-made questionnaire. Beck depression inventory IA is a 21-item questionnaire to assess possible depression and it is validated in Finnish [15]. FIQ is a multidimensional self-administered questionnaire including 10 questions, which evaluate physical functioning, work status, depression, anxiety, sleep, pain, stiffness, fatigue and well-being. It is also validated in Finnish [16]. The self-made questionnaire included questions regarding the pain condition of the patients, the intensity of their current pain, the effect of their pain relief methods, and the rank of FM pain compared to other possible pains.

Those who replied were invited to a clinical visit, where an experienced physician (TH) examined the patients and confirmed the diagnosis of FM according to the criteria of the American College of Rheumatology [17]. Based on careful clinical history and physical examination, including neurological examination with meticulous bedside sensory testing, she also assessed whether the patient had neuropathic pain or not [18]. The level of certainty of the neuropathic pain diagnosis was graded as definite, probable or possible [19]. The grading system has four items: 1. Pain with a distinct neuroanatomically plausible distribution. 2. History suggestive of a relevant lesion or disease affecting the peripheral or central somatosensory system. 3. Demonstration of the distinct neuroanatomically plausible distribution by at least one test. And 4. Demonstration of the relevant lesion or disease by at least one test. The definite grade requires all items 1. to 4. The probable grade requires items 1. and 2. plus either 3. or 4. The possible grade requires items 1. and 2.

### Statistical methods

Results were expressed as means with standard deviation (SD) and 95% confidence intervals (CIs), and as medians with interquartile range (IQR). Statistical comparison between the groups was performed by t-test, permutation test or Chi-square test, when appropriate. Relationships between neuropathic pain and important risk factors were analyzed with univariate and multivariate forward stepwise logistic regression models. Receiver operating characteristic (ROC) curve was constructed to determine the cut-off point of PainDETECT that corresponds to the clinically verified neuropathic pain, with bias corrected bootstrap CIs. Values for the area under the ROC curve from 0.7 to 0.8 indicate reasonable discrimination and values exceeding 0.8 indicate good discrimination. We defined the best cut-off value as the value with the highest accuracy that maximizes the Youden's index. Sensitivity, specificity, positive predictive value, likelihood ratio, Youden's index, and their 95% CI values were calculated.

### Ethical aspects

The study protocol was approved by The Committee of Research Ethics of Central Finland Health Care District, and the patients gave their informed consent in writing.

## Results

**Figure 1:**
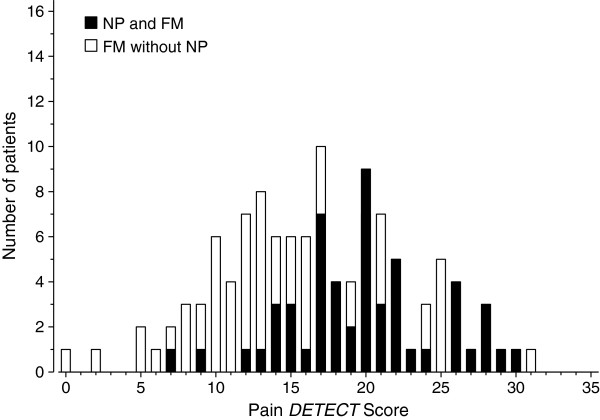
**Distribution of the Pain*DETECT *scores according to the presence or absence of neuropathic pain diagnosis in FM patients.**

The postal survey was mailed to 239 patients with primary FM, and 169 patients (71%) replied. Five patients declined to attend the clinical visit due to long distances. Six patients had filled in questionnaires insufficiently and were excluded from the analyses. Hence 158 patients who had undergone clinical evaluation and had completed the questionnaires adequately were included in the analyses. All of them fulfilled the diagnostic criteria of primary FM. In addition to FM, clinically verified neuropathic pain was diagnosed in 53/158 [34% (95% Cl: 26 to 41)] patients. Neuropathic pain was definite in 16 (10%) patients, probable in 30 (19%) patients and possible in 7 (4%) patients. The Pain*DETECT* score and the intensity of current pain were significantly higher in the patients with neuropathic pain compared to those without it. FM pain was regarded as the worst current pain in 70% of the patients without neuropathic pain and in 41% of the patients with neuropathic pain (p < 0.001) (Table 1, Figure 1). The neuropathic pain diagnoses of the 46 patients with definite or probable neuropathic pain are listed in Table 2.

**Table 1:** Demographic and clinical data of 158 FM patients with and without neuropathic pain diagnosis

**Variables**	**NP N = 53**	**NNP N = 105**	**P-value**
Females, number (%)	49 (92)	100 (95)	0.48
Age, mean (SD)	49 (8)	46 (12)	0.074
BMI, mean (SD)	29.6 (6.6)	27.3 (6.6)	0.041
Duration of diagnosis, median (IQR)	4 (2.7)	3 (2.8)	0.53
FIQ, mean (SD)	52.3 (19.4)	48.0 (20.1)	0.19
Pain*DETECT* score, mean (SD)	19.8 (5.0)	15.9 (5.8)	<0.001
Beck Depression Inventory, mean (SD)	14.4 (7.2)	13.5 (9.4)	0.57
Efficacy of pain relief*, mean (SD)	6.3 (1.7)	6.8 (1.8)	0.13
Health score**, mean (SD)	5.8 (2.1)	5.8 (2.0)	0.95
Current pain intensity***, mean (SD)	6.3 (2.0)	5.6 (2.2)	0.033
Number of patients with FM pain as their worst current pain (%)	22 (41)	73 (70)	<0.001

NP = neuropathic pain, NNP = non-neuropathic pain, BMI = body mass index, FIQ = Fibromyalgia Impact Questionnaire.

*Expressed as numerical rating scale (0 = no pain relief, 10 = maximal pain relief).

** Expressed as numerical rating scale (0 = poor health, 10 = maximal state of health).

*** Expressed as numerical rating scale (0 = no pain, 10 = worst imaginable pain).

**Table 2:** Neuropathic pain diagnoses in 46 patients with probable or definite neuropathic pain

**NP diagnoses**	**Probable NP N = 30**	**Definite NP N = 16**	**Method(s) of confirming definite NP diagnosis**
Lumbar radiculopathy	20	8	ENMG (4), lumbar MRI (4)
Thoracic radiculopathy	1		
Cervical radiculopathy	2	1	cervical MRI
Painful polyneuropathy	0	2	ENMG (2), gene test* (1)
Peripheral nerve entrapment #	4	3	ENMG
Peripheral nerve lesion ¤	3	2	ENMG

NP = neuropathic pain.

* confirming the diagnosis of hereditary polyneuropathy.

# compression of median nerve in 3 and ulnar nerve in 2, tarsal tunnel syndrome in 1.

¤ lesion of peroneal nerve 2, ulnar nerve in 1, cervical plexus in 1 and trigeminal nerve in 1.

**Figure 2:**
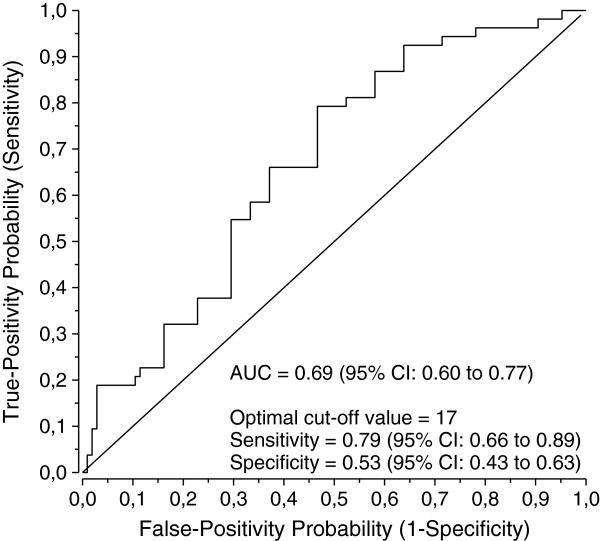
**ROC curve of Pain*DETECT *scores when used to define the presence of neuropathic pain.**

The ROC curve achieved a maximum Youden´s index at score of 17 when sensitivity was 0.79 (95% Cl: 0.66 to 0.89) and specificity 0.53 (95% Cl: 0.43 to 0.63). The area under the curve was 0.69 (95% Cl: 0.60 to 0.77). The predictive value of a positive test and likelihood ratio (positive) were 0.46 (95% Cl: 0.36 to 0.57) and 1.7 (95% Cl: 1.33 to 2.17) respectively (Table 3, Figure 2).

**Table 3:** **Characteristics with 95 per cent confidence intervals of performance of different Pain *DETECT *cut-off scores**

**Characteristics**	**Pain*DETECT*screening**		
	**Cut-off score**		
	**12**	**17**	**19**
Sensitivity	0.94 (0.84 to 0.99)	0.79 (0.43 to 0.89)	0.58 (0.44 to 0.72)
Specificity	0.29 (0.20 to 0.38)	0.53 (0.43 to 0.63)	0.67 (0.57 to 0.76)
Predictive value of positive test	0.40 (0.31 to 0.49)	0.46 (0.36 to 0.57)	0.47 (0.35 to 0.60
Likelihood ratio (positive)	1.32 (1.14 to 1.53)	1.70 (1.33 to 2.17)	1.75 (1.23 to 2.50)
Youden's index	0.23 (0.13 to 0.33)	0.33 (0.17 to 0.47)	0.25 (0.08 to 0.41)

The Pain*DETECT* total score (OR: 1.14 95% Cl: 1.06 to 1.22), FM as the worst current pain (OR: 0.31; 95% 0.16 to 0.62), body mass index (BMI) (OR: 1.05; 95% Cl: 1.00 to 1.11) and the intensity of current pain (OR: 1.20; 95% Cl: 1.01 to 1.41) were significantly associated with the presence of neuropathic pain in univariate analyses. The Pain*DETECT* score and the patient’s own assessment of FM pain as their worst pain entered into the forward logistic regression model (Table 4).

**Table 4:** Logistic regression models for the odds to presence of neuropathic pain in FM patients

**Variable**	**Univariate OR (95% CI)**	**P value**	Multivariate* OR (95% CI)	**P value**
Female gender	0.61 (0.16 to 2.38)	0.48		
Age	1.03 (1.00 to 1.07)	0.076		
Body mass index	1.05 (1.00 to 1.11)	0.048		
Duration of fibromyalgia diagnosis	1.02 (.96 to 1.08)	0.59		
FIQ	1.12 (.94 to 1.33)	0.19		
PainDETECT total score	1.14 (1.06 to 1.22)	<0.001	1.16 (1.08 to 1.25)	<0.001
Beck Depression Inventory	1.01 (.97 to 1.05)	0.56		
Efficacy of pain relief	0.86 (.71 to 1.04)	0.12		
Health score	0.99 (.84 to 1.17)	0.92		
Current pain intensity	1.20 (1.01 to 1.41)	0.034		
FM pain as the worst current pain	0.31 (.16 to .62)	<0.001	0.25 (0.11 to 0.53)	<0.001
IPAQ	1.00 (1.00 to 1.00)	0.56		

*Forward selection. Only those variables are shown which entered the model.

## Discussion

Our main finding showed that Pain*DETECT* cannot distinguish neuropathic pain from non-neuropathic pain in FM patients. In the Pain*DETECT* validation study a cut-off value of 19 points had both sensitivity and specificity of 0.84 [12], whereas that cut-off value reached sensitivity of 0.59 and specificity of 0.67 in our cohort. In our study the optimal cut-off value was 17 points with sensitivity of 0.79 and a low specificity of 0.53. The Pain*DETECT* score and FM as the worst current pain had the strongest association with the presence of neuropathic pain, the latter having negative association, i.e., FM pain as the worst current pain proved to be a protective marker to neuropathic pain. Based on these results of sensitivity and specificity we do not recommend the use of Pain*DETECT* for patients with FM as the principal diagnostic tool. It is worth noting that FM patients were excluded from the validation studies of Pain*DETECT*[12] and DN4 (another neuropathic pain screening tool) [20]. Likewise patients with “mixed pain” were excluded from the validation study of LANSS [21]. A recent review recommended that neuropathic pain screening tools should not be used for patients with widespread pain [22]. Although neuropathic pain screening tools have not been validated with FM patients, Rhem et al. [4] and Koroschetz et al. [23] used Pain*DETECT* to classify FM patients on the basis of their sensory symptoms. On the basis of LANSS results in FM patients Martinez-Lavin et al. [24] even suggested that FM might be a neuropathic pain state.

Although patients with previously diagnosed neuropathic pain were excluded, and therefore the prevalence of neuropathic pain was underestimated in our study, neuropathic pain was found in 37% of the patients. This is about five-fold compared to the prevalence in the general population according to the studies using LANSS [6] and DN4 [7].

It is important to bear the possibility of neuropathic pain in mind when a FM patient complains of sensory symptoms and pain with neuroanatomically plausible distribution. In such cases, careful examination of the patient is needed to support or exclude the diagnosis of neuropathic pain; FM and neuropathic pain are not mutually exclusive. Depending of the location of the pain and findings in the bedside examination, additional test (e.g., electroneuromyography in suspected peripheral nerve lesion, quantitative sensory testing and skin biopsy in suspected small fiber neuropathy, or magnetic resonance imaging in suspected central nervous system lesion) may be indicated (see more detailed report in [10,18]).

It is generally assumed that the bodily distress disorders are highly associated with emotional distress, e.g., mood disorder. However, according to the BDI results, our patients with and without neuropathic pain had similar level of depressive symptoms.

The limitations of our study are retrospective setting, cross-sectional clinical evaluation, descriptive nature of the study (we were not allowed to perform additional diagnostic procedures to improve the level of certainty of the neuropathic pain diagnosis), secondary health care setting, and ignorance of other pains but FM and neuropathic pain.

Diagnosis of neuropathic pain may give an opportunity for curative treatment of the cause of neuropathic pain (e.g., surgical release of a nerve entrapment). In addition, patients with both FM and neuropathic pain might be favorable candidates for a treatment trial with tricyclic antidepressants, pregabalin or duloxetine, which have proved their efficacy for both conditions [25-27].

## Conclusion

This study highlights the importance of thorough clinical examination when a FM patient emerges with new symptoms. Neuropathic pain screening tools such as Pain*DETECT* are not as useful for FM patients as for other patient. However, if it is used, a positive Pain*DETECT* score still attracts the clinician’s attention to the possibility of neuropathic pain and encourages performing an adequate neurological examination and the consideration of further testing when needed.

## Competing interests

The authors do not have any competing interests in this study.

## Authors’ contributions

JG, HK and MH conceived of the study and did the statistical analyzes. TH was responsible of clinical examination of participants. JG was responsible of writing the manuscript. PH contributed to study design. HK, PH and MH critically reviewed the manuscript. All authors read and approved the final manuscript.

## Pre-publication history

The pre-publication history for this paper can be accessed here:

http://www.biomedcentral.com/1471-2377/13/21/prepub
